# A Pattern Reconfigurable Antenna Using Eight-Dipole Configuration for Energy Harvesting Applications

**DOI:** 10.3390/s23208451

**Published:** 2023-10-13

**Authors:** Mohamed Aboualalaa, Hesham A. Mohamed, Thamer A. H. Alghamdi, Moath Alathbah

**Affiliations:** 1 Microstrip Department, Electronics Research Institute, Cairo 11843, Egypt; hesham_280@eri.sci.eg; 2 Wolfson Centre for Magnetics, School of Engineering, Cardiff University, Cardiff CF24 3AA, UK; 3 Electrical Engineering Department, School of Engineering, Albaha University, Albaha 65779, Saudi Arabia; 4 Department of Electrical Engineering, College of Engineering, King Saud University, Riyadh 11421, Saudi Arabia; malathbah@ksu.edu.sa

**Keywords:** dipole antenna, electronically steering, energy harvesting, reconfigurable antenna, RF switch matrix

## Abstract

A pattern reconfigurable antenna, composed of eight elements, is proposed for energy harvesting applications. Pattern reconfigurable antennas are a promising technique for harvesting from different wireless sources. The radiation pattern of the proposed antenna can be steered electronically using an RF switch matrix, covering an angle range from 0 to 360 degrees with a step size of 45 degrees. The proposed antenna primarily consists of an eight-dipole configuration that shares the same excitation. Each dipole is excited using a balun comprising a quarter-wavelength grounded stub and a quarter-wavelength open-circuit stub. The proposed antenna operates in the frequency range of 4.17 to 4.5 GHz, with an impedance bandwidth of 7.6%. By switching between the different switches, the antenna can be steered with a narrower rotational angle. In addition, the antenna can work in an omnidirectional mode when all switches are in the ON state simultaneously. The results demonstrate a good agreement between the numerical and experimental findings for the reflection coefficient and radiation characteristics of the proposed reconfigurable antenna.

## 1. Introduction

Reconfigurable antennas are a type of antenna that can change their operating parameters, such as frequency, polarization, radiation pattern, and impedance, in real-time or under specific conditions [1,2,3,4,5,6,7,8]. This reconfigurability allows the antenna to adapt to different communication standards, frequency bands, or user requirements. Reconfigurable antennas are gaining popularity due to their flexibility and potential to enhance the performance of wireless communication systems. They can improve network coverage, increase the data rate, reduce interference, and provide seamless connectivity. This technology has significant implications for emerging applications, such as the Internet of Things (IoT), 5G networks, and satellite communication.

Pattern reconfigurable antennas are capable of dynamically changing their radiation patterns to accommodate varying operational requirements [9,10,11,12]. This type of antenna holds the advantage of electronically adjusting its radiation beams in different directions, eliminating the need for physical movement. With their ability to adapt to different directions, pattern reconfigurable antennas are gaining popularity in wireless communication systems, offering a flexible solution for improving network coverage, enhancing signal quality, and mitigating interference.

In this study, we propose a design for a pattern reconfigurable antenna specifically tailored for energy harvesting applications. Energy harvesting involves converting ambient energy from the surrounding environment, such as electromagnetic waves, into electrical energy that can power small electronic devices [13,14,15,16,17,18,19,20]. Consequently, reconfigurable antennas offer several advantages over conventional antennas when it comes to energy harvesting. They can be optimized to efficiently harvest energy from various sources and frequencies. By adjusting the antenna’s radiation pattern, polarization, or frequency response, the harvested energy can be maximized even in dynamic and varying environments. Moreover, reconfigurable antennas can be integrated with other energy harvesting components, including rectifiers, impedance matching networks, and energy storage devices, to form a comprehensive energy harvesting system. Considering these aspects, the utilization of reconfigurable antennas in energy harvesting applications has the potential to revolutionize wireless power transfer and facilitate the development of self-powered and autonomous devices.

**Figure 1:**
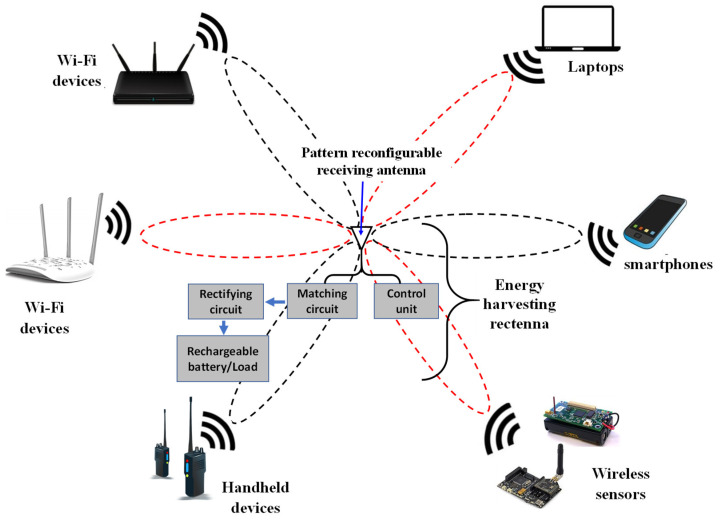
Configuration of energy harvesting from different RF sources.

Multiple antennas are typically required to harvest from different RF sources [21,22,23], which results in a larger system size. However, a pattern reconfigurable antenna offers a suitable solution for harvesting energy from various sources located at different positions. Depending on the signal intensity, the antenna can steer its direction using a dedicated DSP control unit. The operational concept of using a pattern reconfigurable antenna for energy harvesting is demonstrated in Figure 1. The reconfigurable antenna is connected to a control unit, which in turn can determine the direction in which the antenna operates. Additionally, the antenna is connected in parallel with a rectifying circuit through a matching circuit, forming the rectenna structure. This rectenna can be connected to a rechargeable battery or directly to a load. Reconfigurable antennas hold great promise for achieving reliable and efficient energy harvesting systems. In this study, we focused on the receiving pattern reconfigurable antenna and its electronic steering capability. To the best of our knowledge, the proposed structure is the first to suggest a steerable pattern in energy harvesting applications for scavenging from various RF sources.

## 2. Antenna Configuration

**Figure 2:**
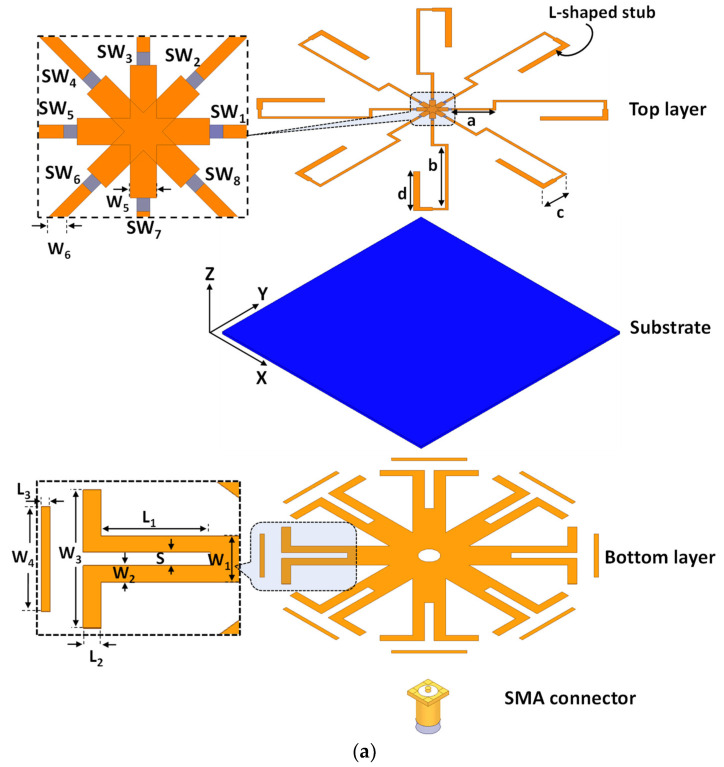

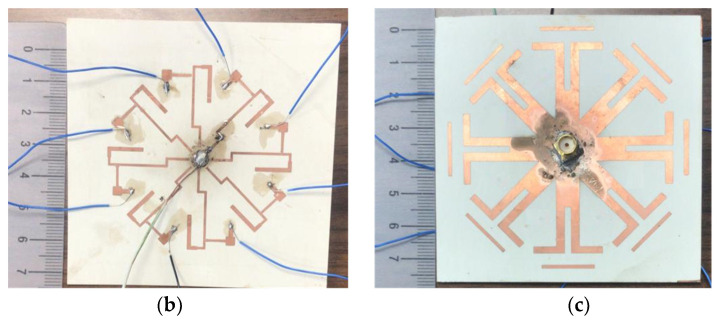
(**a**) The 3D assembly configuration of the proposed antenna, (**b**) top view of the antenna’s fabricated prototype, and (**c**) bottom view of the antenna’s fabricated prototype; a = 7 mm, b = 17 mm, c = 4 mm, d = 10.5 mm, S = 2 mm, L_1_ = 12 mm, L_2_ = 2 mm, L_3_ = 1 mm, W_1_ = 7 mm, W_2_ = 2.5 mm, W_3_ = 21 mm, W_4_ = 16 mm, W_5_ = 1 mm, and W_6_ = 0.5 mm.

**Figure 3:**
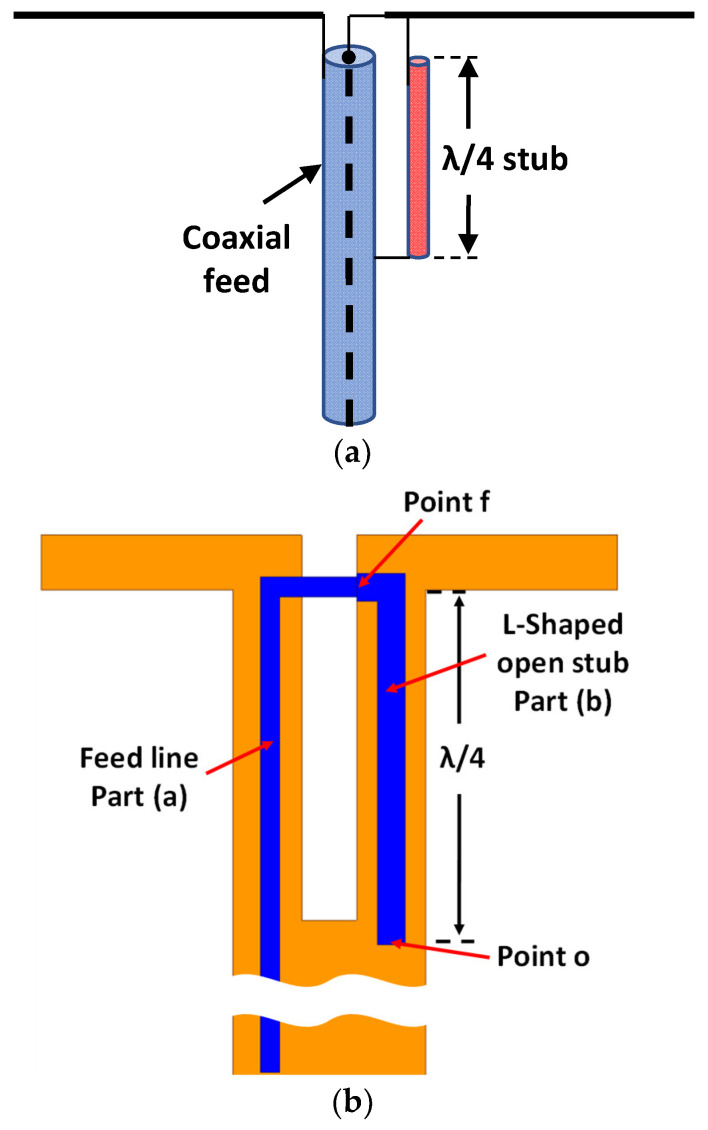
Configuration of (**a**) coaxial balun structure, and (**b**) balun used with the proposed antenna.

Figure 2a illustrates the configuration of the proposed antenna design. It comprises an eight-dipole configuration designed on the ground plane (bottom layer). The dipoles are connected to a common feeding point, and each dipole has a director to provide a better directional characteristic of the antenna. On the opposite side of the substrate, the top layer has eight open-circuited stubs positioned to excite the dipoles. Each stub excites a corresponding dipole through a coaxial probe feed. Photographs showing the top and bottom views of the fabricated prototype for the proposed antenna are displayed in Figure 2b,c. A balun, depicted in Figure 3a [24], is utilized for feeding the different dipoles. However, in the proposed design, we deviate from directly connecting the feed line to the other terminal of the dipole. Instead, we incorporate a λ/4 L-shaped open-circuited stub, which provides greater flexibility in adjusting the resonance frequency for the dipole structure. Consequently, the feeding structure comprises two parts, as illustrated in Figure 3b. Part (a) represents the feed line, with a width of 0.5 mm, utilized for exciting the dipole. Part (b) consists of an L-shaped open stub with a double width of the part (a), which connects the feed line to the other terminal of the dipole antenna. At the open-ended section of the L-shaped stub (at point o), the impedance is infinity. Thus, at a distance of λ/4 from this open-end point, the stub functions as a short circuit with the ground plane, connecting the feed line (at point f) with the other terminal of the dipole antenna on the ground plane.

In Figure 2, eight switches (SW_1_, SW_2_, SW_3_, SW_4_, SW_5_, SW_6_, SW_7_, and SW_8_) are strategically placed along the feeding lines to enable pattern reconfigurability. Initially, during the simulation, the RF HPND-4005 diode series was employed to achieve the desired reconfigurability. To replicate the behavior of physical diodes, an equivalent circuit model consisting of a series R-L circuit in the ON state and a parallel R-C circuit in the OFF state was utilized. However, for the actual measurements, an RF switch matrix (Mini-Circuits USB-8SPDT-A18 [25]) with low insertion loss (0.2 dB) and high isolation (85 dB) was employed. Therefore, we substituted the equivalent circuit model with the S2P files of the switch matrix during the full-wave simulation analysis, yielding nearly identical results.

The proposed antenna was fabricated on a thin Rogers RT/duroid 6002^TM^ substrate, featuring a thickness (h) of 0.76 mm, a dielectric constant (ε_r_) of 2.94, a dielectric loss tangent (tanδ) of 0.0012, and a copper thickness (t) of 0.0035 mm. The optimized geometrical parameters of the proposed antenna are listed in the caption of Figure 2.

**Figure 4:**
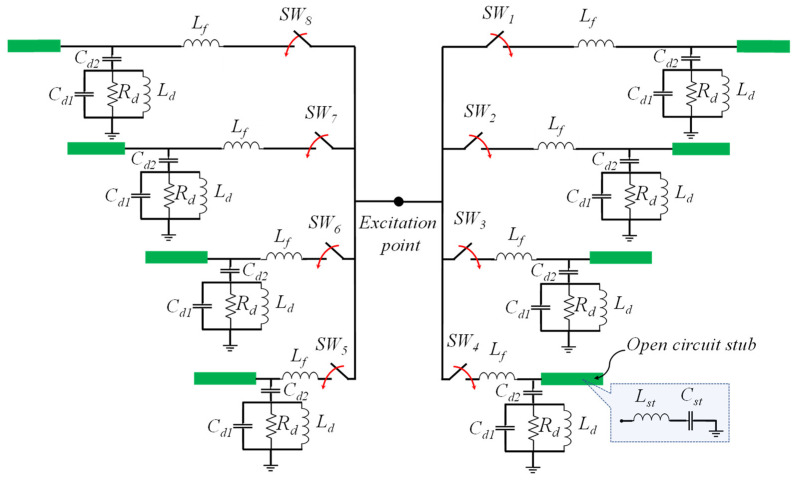
Equivalent circuit model of the proposed antenna structure.

An equivalent circuit model is illustrated in Figure 4 to provide a more detailed explanation and is proposed to simplify the operation of the proposed antenna. Initially, the excitation is distributed among eight branches, each equipped with an RF switch. The feed line is symbolized with an inductor denoted as *L_f_*. Additionally, we adopted a four-element equivalent circuit model [26] to represent the dipole antenna, which consists of a parallel connection involving *R_d_*, *L_d_*, and *C_d1_* arranged in series with *C_d2_*. Furthermore, each λ/4 open-circuit stub, linked to every feed line, can be effectively described as a series *LC* circuit (*L_st_*, and *C_st_*), as depicted in Figure 4.

## 3. Results and Discussion

### 3.1. Reflection Coefficient Results

**Figure 5:**
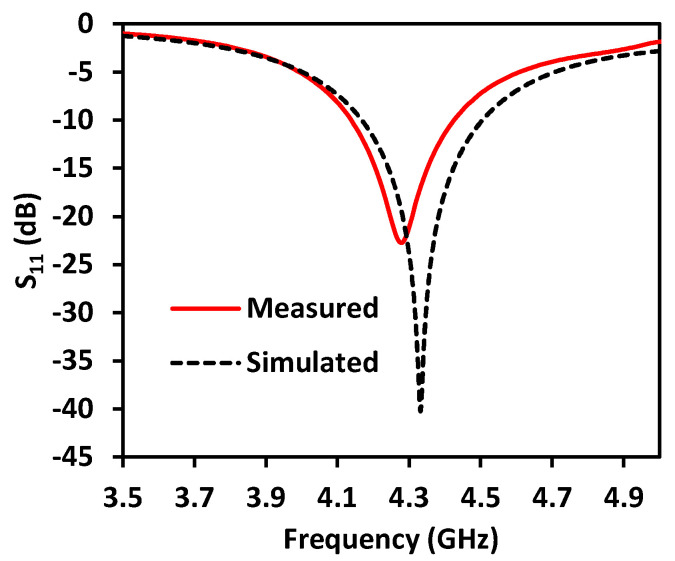
Numerical and experimental findings of the proposed pattern reconfigurable antenna when SW_1_ is in ON state while the other switches are in OFF state.

**Figure 6:**
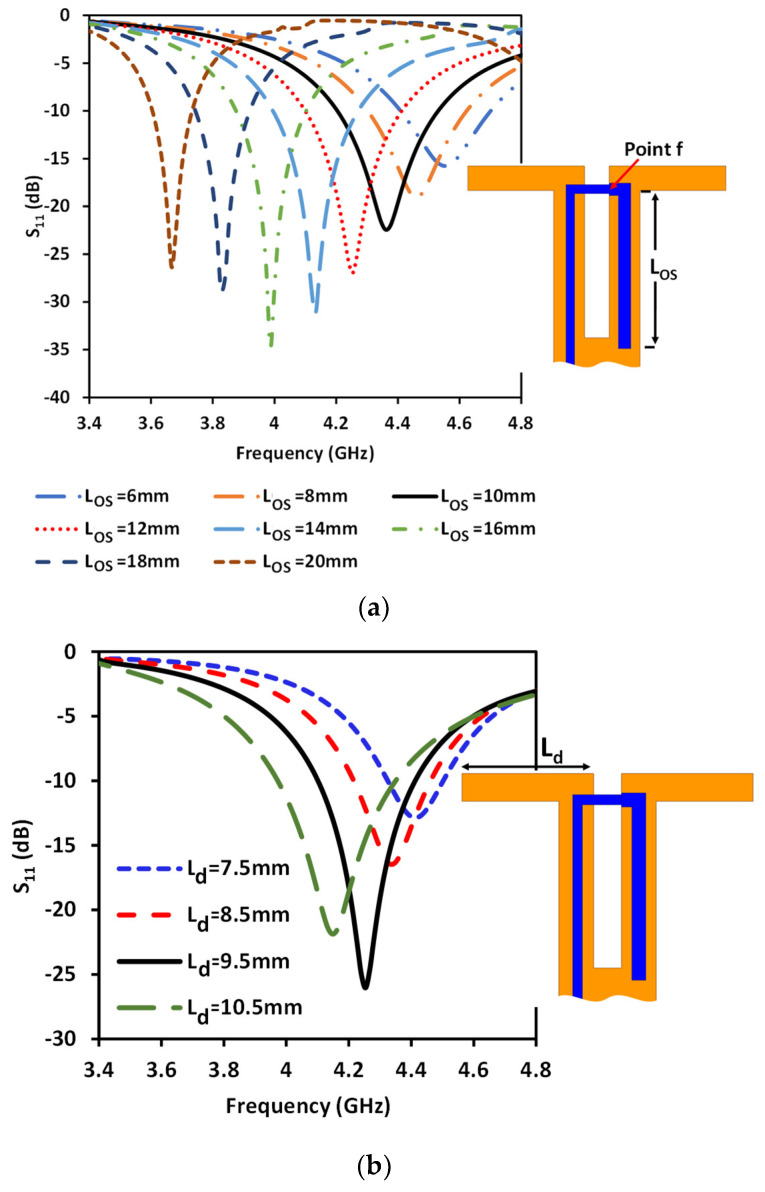

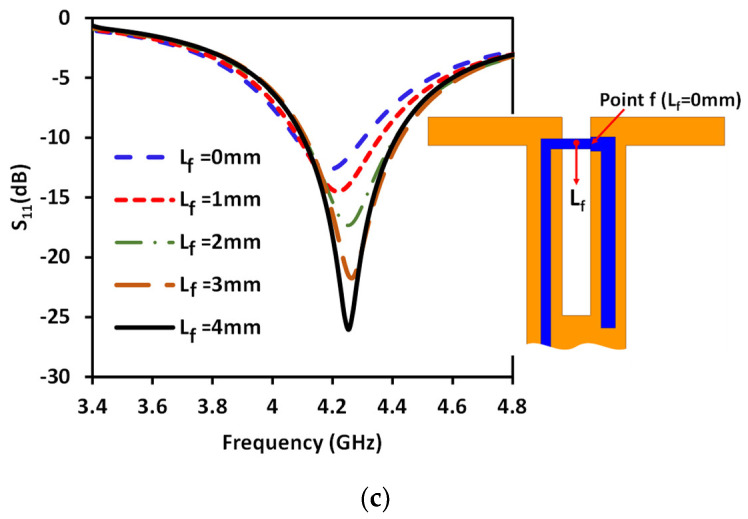
S_11_ parametric study for (**a**) different lengths of λ/4 open-circuit stub (L_OS_), (**b**) different lengths of the dipole (L_d_), and (**c**) different positions of the feed point.

To verify the reconfigurability property of the proposed antenna, the antenna was designed and optimized using a full-wave analysis simulation (ANSYS High Frequency Structure Simulator (HFSS)). Subsequently, the antenna was fabricated and measured to validate the simulation results. Figure 5 shows a comparison between the numerical and experimental results of the antenna’s reflection coefficient when SW_1_ is in the ON state, while the remaining switches are in the OFF state. Notably, the proposed antenna exhibited consistent reflection coefficient characteristics during pattern steering across various dedicated radiation directions. For this study, a frequency range of 4 to 4.5 GHz within the sub-6 GHz band was selected as the operational range. Nonetheless, the antenna parameters can be adjusted to accommodate different frequency bands. The proposed antenna operates within the range of 4.17 to 4.5 GHz, offering an impedance bandwidth of 7.6%. Parametric studies were performed to investigate the impact of various antenna parameters, such as the length of the λ/4 L-shaped open stub, dipole length, and feeding location, as depicted in Figure 6.

**Figure 7:**
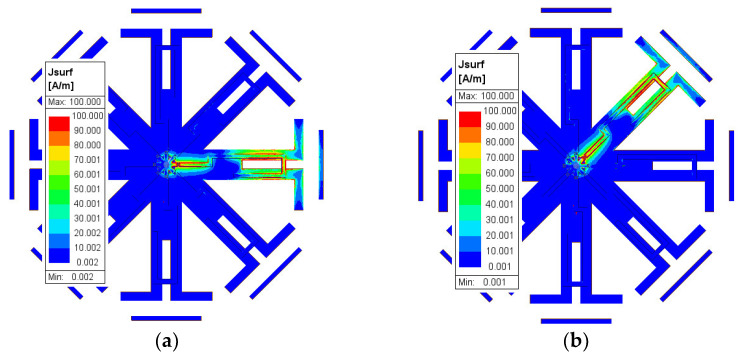

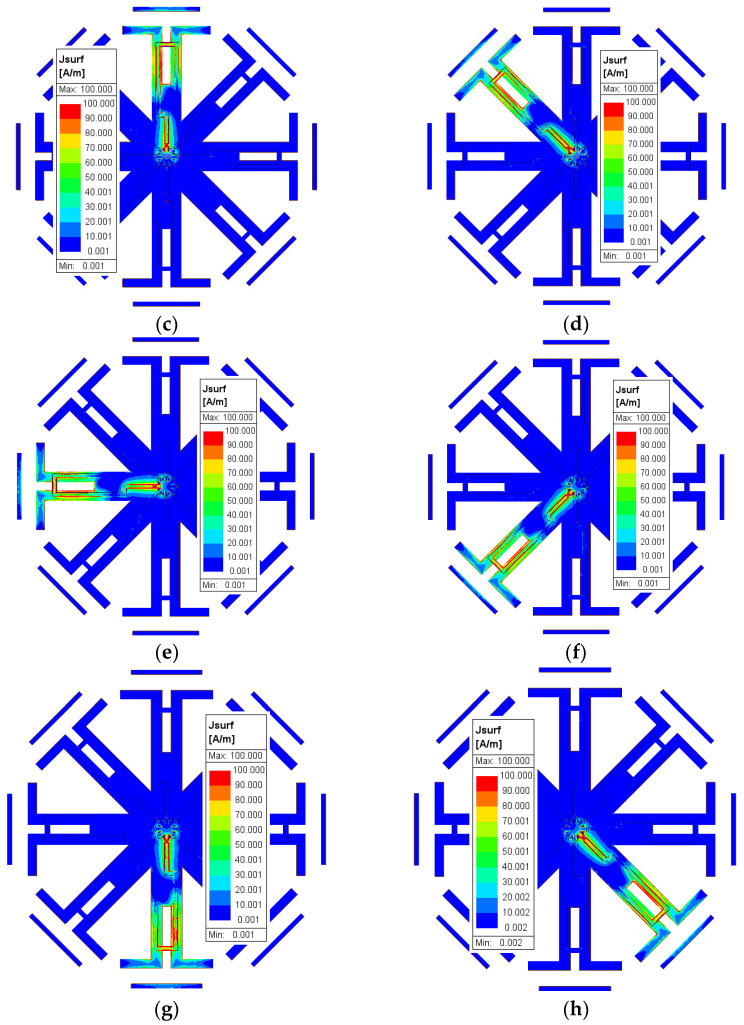
Surface current distribution when (**a**) SW_1_ is ON, (**b**) SW_2_ is ON, (**c**) SW_3_ is ON, (**d**) SW_4_ is ON, (**e**) SW_5_ is ON, (**f**) SW_6_ is ON, (**g**) SW_7_ is ON, and (**h**) SW_8_ is ON.

By adjusting the length of the λ/4 open-circuit stub (*L_OS_*), the matching can be achieved at different values of the operating frequency of the dipole, as shown in Figure 6a. When the stub length is shorter, it achieves the matching at a higher frequency for the dipole antenna. Conversely, increasing the stub length allows for matching adjustment at a lower frequency. Furthermore, the operating frequency can be adjusted by controlling the length of the dipole (L_d_), as displayed in Figure 6b. Additionally, altering the feed position has an impact on the input impedance and consequently the antenna matching. The study of the feed location started when the feed point was at dipole as a reference position (when the feeding is at point f, i.e., L_f_ = 0 mm), shown in Figure 6c. Subsequently, the feeding position shifted away from the dipole towards the excitation position. This shift has a slight effect on the resonance frequency, as demonstrated in Figure 6c, where the currents in the two parallel lines of the ground plane counterbalance each other. However, it primarily influences the matching, as the input impedance changes with the alteration of the feed location. Therefore, the operating frequency and antenna matching can be adjusted by manipulating these parameters (L_OS_, L_d_, and L_f_). As shown in Figure 6a, by adjusting the length of the open stub we can tune the frequency of the proposed antenna from approximately 3.7 GHz to 4.6 GHz. This frequency range falls within the proposed sub-6 5G spectrum for several countries, such as the US (3.7–3.98 GHz), Canada (3.65–4 GHz), South Korea (3.7–4 GHz), and Japan (3.6–4.1 GHz) [27]. We conducted our study around 4.3 GHz; however, the frequency can be easily adjusted to different bands. Figure 7 presents the surface current distribution for different states of the switches, revealing the concentration of current at each branch when its corresponding switch is in the ON state.

**Figure 8:**
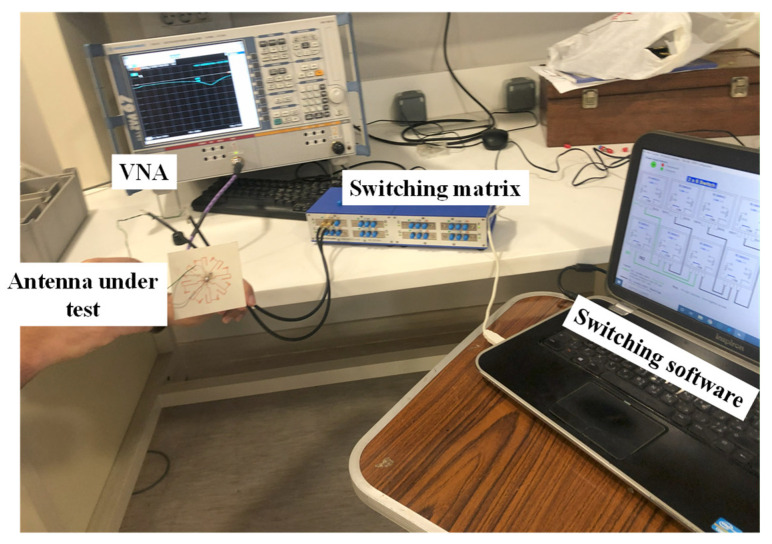
Measurement setup for antenna reflection parameters.

**Figure 9:**
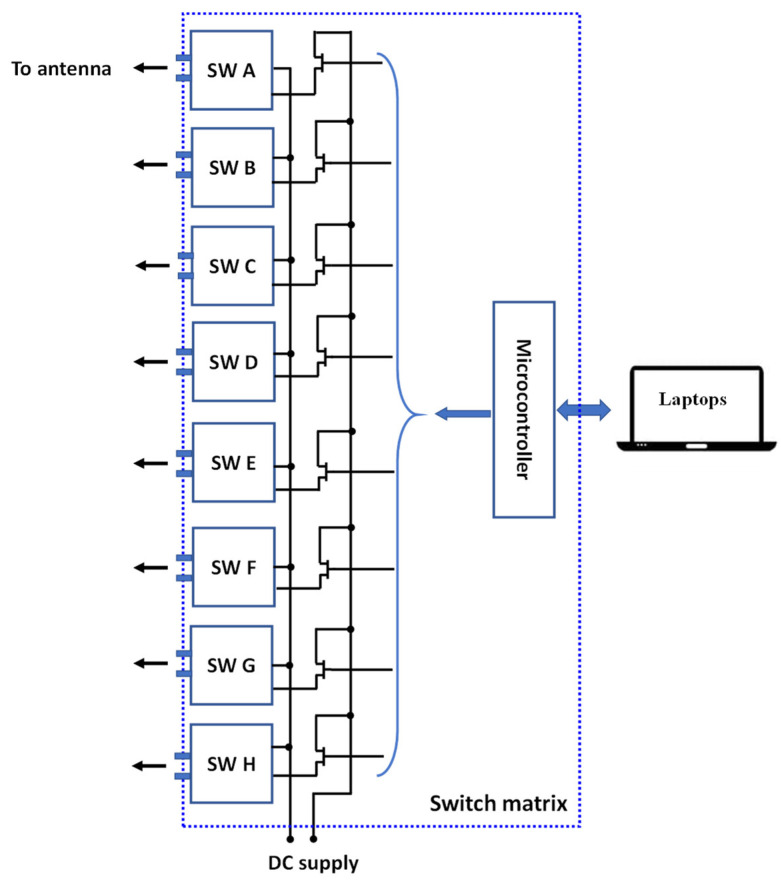
Circuit diagram of the switch matrix.

**Figure 10:**
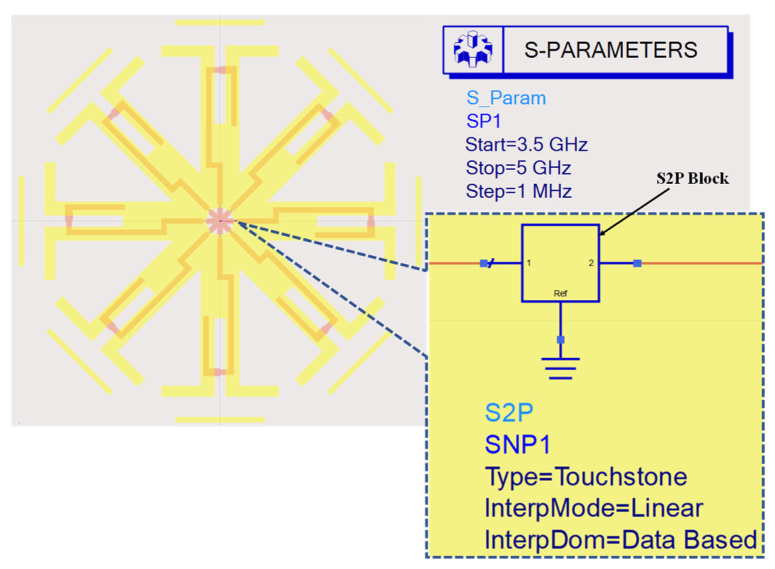
ADS model used for conducting a co-simulation between the schematic and momentum.

During the measurements, we utilized the Mini-Circuits USB-8SPDT-A18 RF switch matrix, as depicted in Figure 8. This switch matrix is capable of operating from DC to 18 GHz and offers low insertion loss (0.2 dB) and high isolation (85 dB). It served a dual function, both enabling ON/OFF switching operation and functioning as the control unit for the electronic steering process. With this switch matrix, we can electronically switch between different directions of the main beam of the proposed reconfigurable antenna, providing flexibility and adaptability to the system. Below is a summary of the sequence for performing the simulation and measurements:

- We initiated the simulation by employing an equivalent circuit model of a PIN diode (HPND4005) [6].
- During the measurements, we utilized a switch matrix instead of soldering the PIN diodes. In this new scheme, the switch matrix replaced the use of PIN diodes, and the antenna was connected to the switch matrix using only connecting wires. Figure 9 shows the circuit diagram of the switch matrix, illustrating how the switches were connected.
- In order to consider the effect of using the switch matrix during the simulation, we utilized the S2P files provided by the manufacturing company of the switch matrix. These files characterize both the ON and OFF states of the switch matrix. To achieve this, we removed the PIN diodes and substituted them with S2P blocks at the position between the two terminals of each switch. Each S2P block was used to read the S2P file for its respective switch.
- To incorporate the S2P blocks in the simulation model, we utilized the Keysight ADS simulator by conducting a co-simulation between the schematic and momentum, as illustrated in Figure 10. To switch between the different switching states in the simulation, two S2P files were loaded into the S2P block (one for the ON state and the other for the OFF state). For example, if only SW1 needed to be ON while the others were OFF, we loaded the S2P block assigned to SW1 with the S2P file for the ON state, while the other S2P blocks were loaded with the S2P file for the OFF state.
- On the other hand, to achieve the switching operation during the measurements, the matrix’s software was used to control the states of the switches.

### 3.2. Radiation Characteristics

The numerical and measurement results for the 2D normalized radiation patterns (E-plane and H-plane) of the proposed reconfigurable antenna, under various switch configurations, are provided in Table 1 and Table 2. In Table 1, the radiation results when switches SW_1_, SW_2_, SW_3_, and SW_4_ are in the ON position are displayed. Meanwhile, Table 2 showcases the results for the remaining four cases, where switches SW_5_, SW_6_, SW_7_, and SW_8_ are in the ON position. The graphs clearly demonstrate that by altering the states of the switches, a rotatable radiation pattern can be obtained. Through control of the switching matrix states, the radiation pattern can be steered at angles of 0°, 45°, 90°, 135°, 180°, 225°, 270°, and 315°. The antenna provides an average antenna gain of 4.2 dBi and a radiation efficiency of 80%.

**Figure 11:**
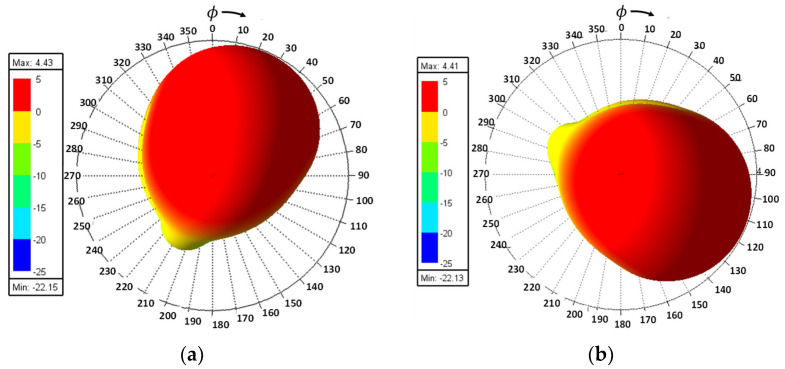
The 3D radiation patterns when: (**a**) SW_1_ and SW_2_ are ON, and (**b**) SW_3_ and SW_4_ are ON.

**Figure 12:**
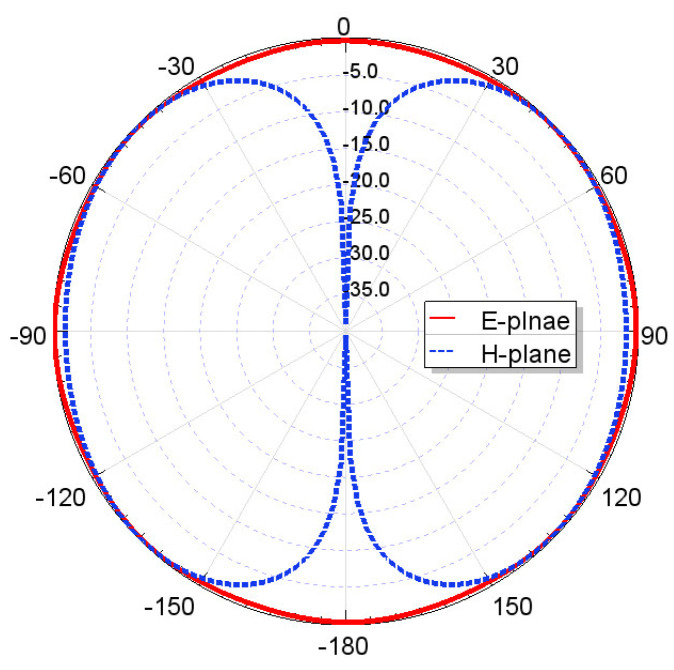
Omnidirectional propagation mode when all switches are in ON state.

**Figure 13:**
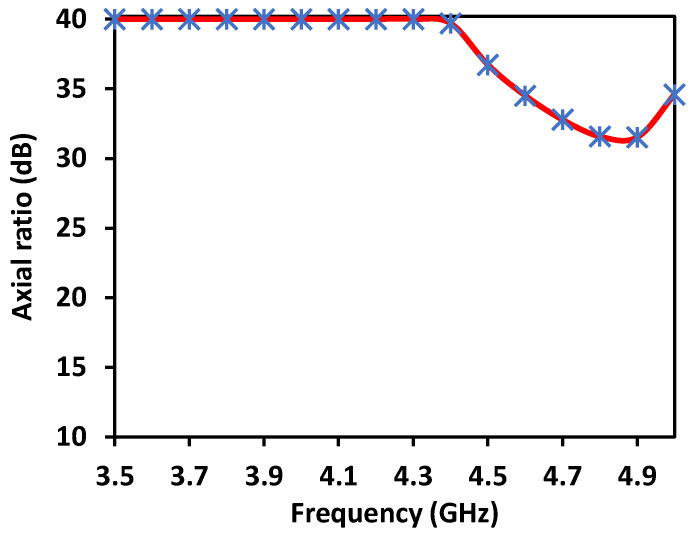
Axial ratio of the proposed antenna.

**Figure 14:**
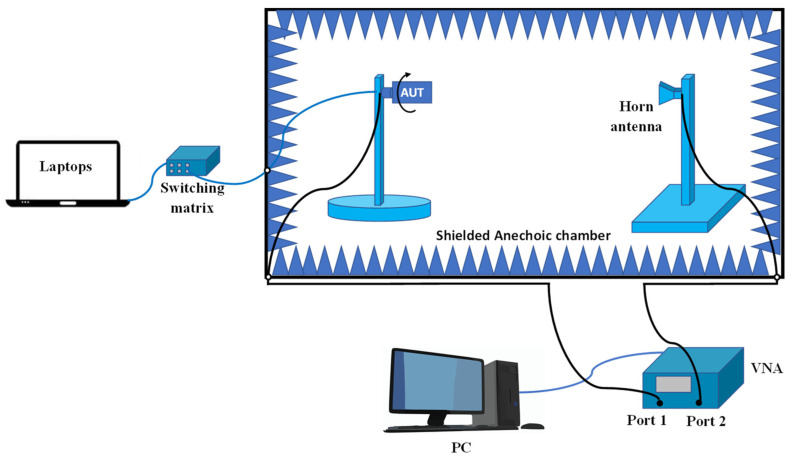
Radiation pattern measurement setup.

Furthermore, by activating more than one switch in the ON state, the radiation pattern can be steered with a smaller angular step. For instance, when both SW_1_ and SW_2_ are simultaneously in the ON state, the main radiation pattern can be directed towards the midpoint between the dipoles of these two switches (at *ϕ* = 22.5°). A case study showcasing the connection of multiple switches is depicted in Figure 11. The figure displays the radiation patterns achieved by connecting SW_1_ and SW_2_, as well as SW_3_ and SW_4_, resulting in radiation patterns directed at *ϕ* = 22.5° and *ϕ* = 112.5°, respectively. Moreover, when all switches are set to the same state, i.e., all switches are simultaneously in the ON state, the antenna operates in an omnidirectional mode, as demonstrated in Figure 12. The proposed dipole antenna exhibits linear polarization characteristics, as depicted in Figure 13. The antenna’s axial ratio values in the main direction of the radiation pattern fall within the range of 30 to 40 dB. The radiation pattern measurement setup that was employed to assess the radiation characteristics of the proposed antenna is illustrated in Figure 14.

## 4. Conclusions

We propose a pattern reconfigurable antenna specifically designed for energy harvesting applications. This antenna possesses the ability to electronically steer at various rotational angles, covering a full 360°, enabling it to effectively scavenge energy from diverse sources located at different positions. To achieve matching, a balun comprising a λ/4 open-circuit stub was employed. The fabricated antenna was validated by verifying its performance at different directions using an RF switch matrix. The comparison between the numerical and measurement results demonstrates a high level of agreement, affirming the accuracy and reliability of the proposed antenna design.

## Figures and Tables

**Table 1:** The 2D normalized polar plots of E-plane and H-plane radiation patterns when switches SW_1_, SW_2_, SW_3_, and SW_4_ are in the ON position.

	Plane	E-Plane	H-Plane
Switch State			
SW_1_ is ON		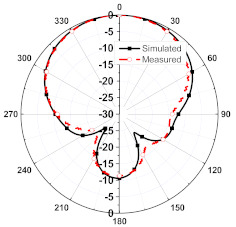	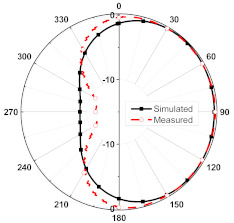
SW_2_ is ON		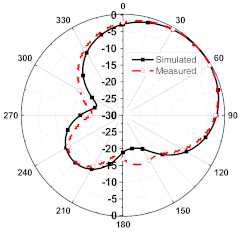	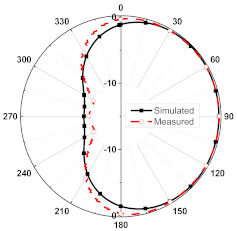
SW_3_ is ON		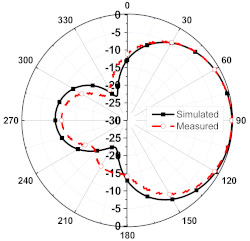	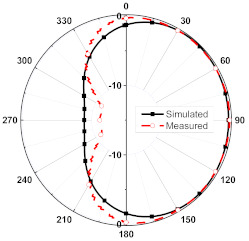
SW_4_ is ON		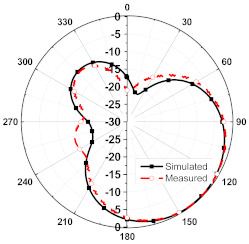	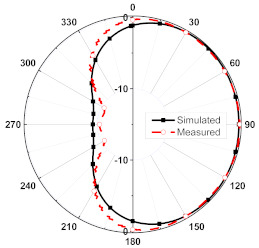

**Table 2:** The 2D normalized polar plots of E-plane and H-plane radiation patterns when switches SW_5_, SW_6_, SW_7_, and SW_8_ are in the ON position.

	Plane	E-Plane	H-Plane
Switch State			
SW_5_ is ON		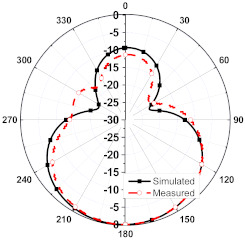	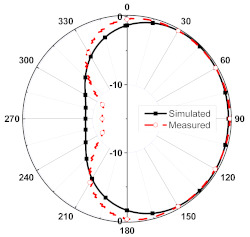
SW_6_ is ON		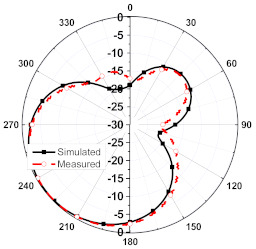	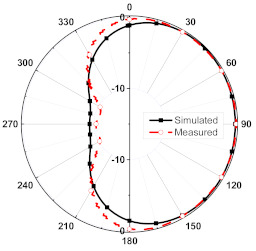
SW_7_ is ON		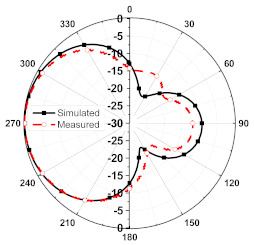	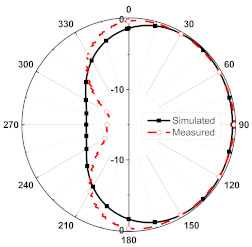
SW_8_ is ON		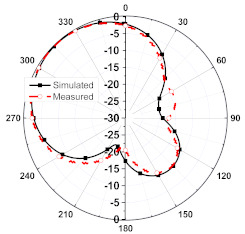	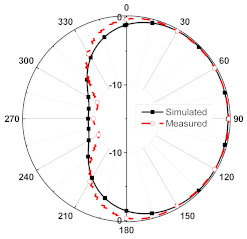

## Data Availability

The data supporting the findings of this study are available upon request from the corresponding author.
